# Supplementary material for: Association Between Dietary Patterns and Cognitive Function in Midlife Adults: The Bogalusa Heart Study
Source: Nutrients. 2025 May 10;17(10):1636. doi: 10.3390/nu17101636 (PMC12113793; doi:10.3390/nu17101636)
Supplement: Supplementary file 1 [file nutrients-17-01636-s001.zip › Supplementary Figure 1_Nutrients.pdf]

Association between Diet Quality and Cognitive Function in Midlife Adults: the Bogalusa Heart Study, Kristen Ogarrio

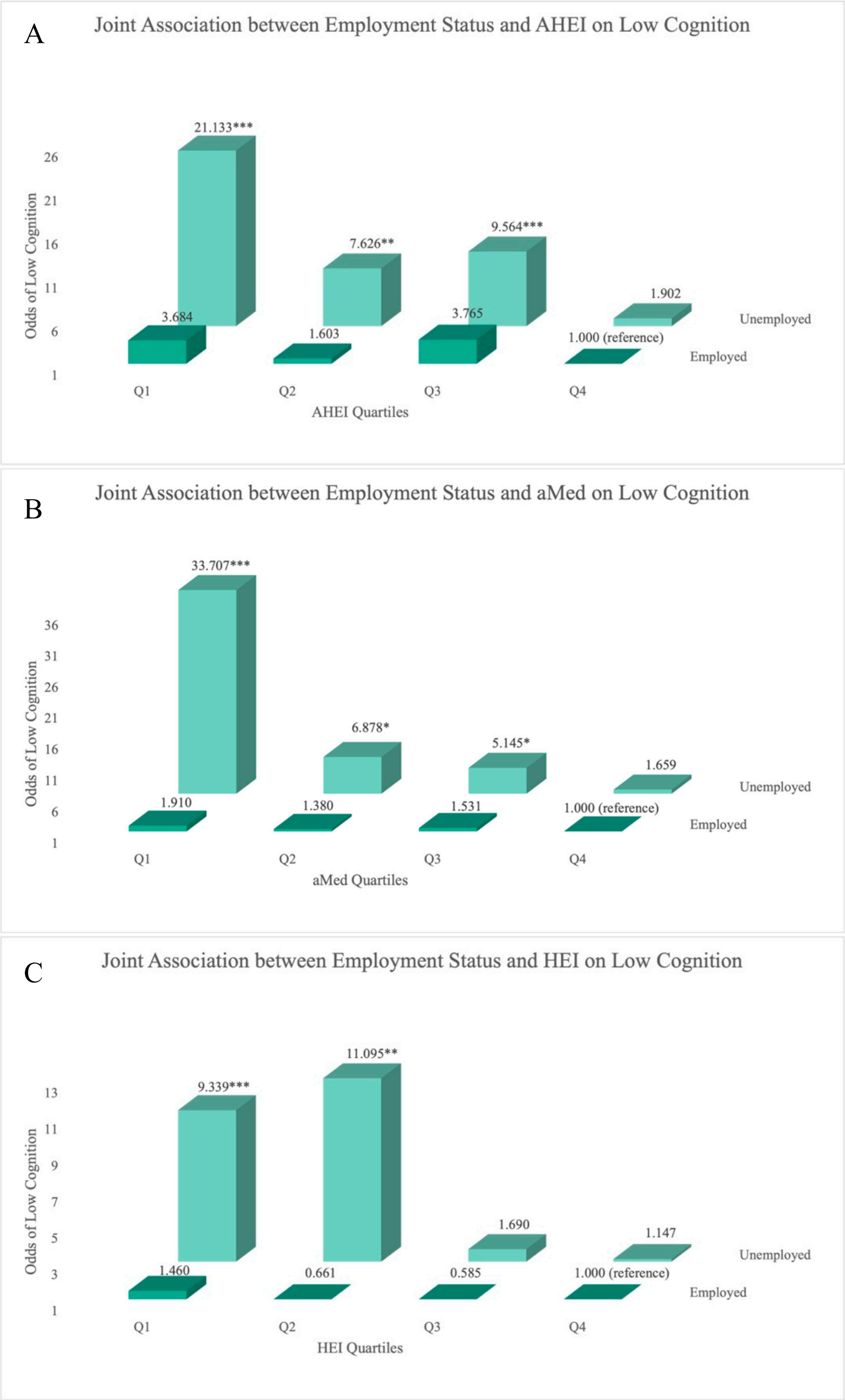

Supplementary Figure S1. Joint effect of diet quality and employment on aOR of low cognitive profile. (A) Adjusted for joint effect of AHEI quartile and employment status, age, sex, race, education, smoking status, total energy intake, MET-minutes, BMI, diabetes, and hypertension. (B) Adjusted for joint effect of HEI quartile and employment status, age, sex, race, education,

smoking status, total energy intake, MET-minutes, BMI, diabetes, and hypertension. (C) Adjusted for joint effect of aMed quartile and employment status, age, sex, race, education, smoking status, total energy intake, MET-minutes, BMI, diabetes, and hypertension.

* $p < 0.05$ ** $p < 0.01$ *** $p < 0.005$
