# Supplementary material for: Association Between Dietary Patterns and Cognitive Function in Midlife Adults: The Bogalusa Heart Study
Source: Nutrients. 2025 May 10;17(10):1636. doi: 10.3390/nu17101636 (PMC12113793; doi:10.3390/nu17101636)
Supplement: Supplementary file 1 [file nutrients-17-01636-s001.zip › Supplementary Tables_nutrients.pdf]

Association between Diet Quality and Cognitive Function in Midlife Adults: the Bogalusa Heart Study, Kristen Ogarrio

Supplementary Table S1. Stratified Least Squares Mean Estimates (95% CI) of Global Cognitive Score Across Dietary Pattern Quartiles (N = 1053)

**Alternate Healthy Eating Index 2010**							
**Variables**	N (%)	Q1[1]	Q2[1]	Q3[1]	Q4[1]	P_{Trend}[2]	$P_{Interaction}$[3]
Age							0.3115
≤ 50 years	594 (56.41)	0.630 (-0.604, 1.864)	1.354 (0.025, 2.682)*	1.260 (-0.241, 2.761)	2.203 (0.377, 2.030)*	0.1099	
> 50 years	459 (43.59)	-0.844 (-2.234, 0.545)	0.599 (-0.0843, 2.042)	-1.554 (-3.022, -0.087)*	0.856 (-0.516, 2.227)	0.1971	
Sex							0.6570
Male	410 (38.94)	-0.494 (-1.783, 0.796)	0.338 (-1.184, 1.859)	-0.949 (-2.462, 0.564)	1.540 (-0.230, 3.309)	0.2263	
Female	643 (61.06)	0.337 (-0.942, 1.616)	1.554 (0.308, 2.801)*	1.127 (-0.300, 2.553)	2.333 (0.937, 3.729)***	**0.0246**	
Race							0.8551
White	723 (68.86)	1.444 (0.518, 2.370)***	2.522 (1.400, 3.643)***	1.279 (0.085, 2.473)*	3.365 (2.041, 4.690)***	0.0602	
Black	327 (31.14)	-1.490 (-3.757, 0.778)	-1.384 (-3.199, 0.431)	-1.631 (-3.658, 0.396)	0.198 (-1.769, 2.165)	0.1811	
Education							0.8497
≤ HS / GED[4]	727 (69.17)	-2.104 (-3.135, -1.073)***	-1.203 (-2.347, -0.058)*	-1.978 (-3.194, -0.762)***	-0.111 (-1.447, 1.225)	0.0500	
> HS / GED[4]	324 (30.83)	2.555 (0.912, 4.199)***	3.740 (2.089, 5.392)***	3.135 (1.315, 4.955)***	5.166 (3.248, 7.083)***	**0.0203**	
Employment Status							0.7098
Employed	695 (66.25)	0.370 (-0.825, 1.566)	0.824 (-0.477, 2.125)	-0.112 (-1.479, 1.256)	2.215 (0.871, 3.559)***	**0.0436**	
Not Employed	354 (33.75)	-0.939 (-2.620, 0.742)	0.845 (-0.779, 2.468)	0.328 (-1.414, 2.071)	0.964 (-1.081, 3.009)	0.1513	
Smoking Status							0.3851
Active Smoker	177 (16.81)	0.333 (-1.687, 2.352)	0.937 (-1.115, 2.990)	0.436 (-2.243, 3.116)	2.149 (-1.227, 5.525)	0.4365	
Not Active Smoker	876 (83.19)	0.270 (-1.305, 0.766)	0.823 (-0.209, 1.855)	-0.035 (-1.096, 1.026)	1.824 (0.715, 2.933)***	**0.0137**	
Total Energy Intake							0.4383
≤ 2700 kcal/d	799 (75.88)	-0.026 (-0.812, 0.760)	0.575 (-0.180, 1.330)	0.277 (-0.503, 1.058)	1.233 (0.354, 1.891)***	**0.0260**	
> 2700 kcal/d	254 (24.12)	0.1582 (-1.164, 1.480)	0.417 (-1.006, 1.840)	-0.162 (-1.622, 1.298)	1.877 (0.230, 3.524)*	0.1528	
Physical Activity[5]							0.5736
Low	622 (59.07)	-1.253 (-2.312, -0.193)*	0.366 (-0.742, 1.474)	-1.368 (-2.549, -0.188)*	0.607 (-0.551, 1.764)	0.0672	
Moderate	113 (10.73)	0.605 (-1.605, 2.814)	-0.071 (-2.847, 2.704)	-0.151 (-2.329, 2.027)	0.863 (-1.822, 3.549)	0.9745	
High	318 (30.20)	-0.694 (-2.154, 0.765)	-0.981 (-2.472, 0.510)	0.433 (-1.173, 2.038)	1.244 (-0.527, 3.015)	**0.0367**	
BMI[6]							0.5738
< 30	499 (47.39)	-0.832 (-2.132, 0.469)	0.970 (-0.415, 2.356)	-0.462 (-2.032, 1.109)	1.959 (0.341, 3.578)*	**0.0116**	
≥ 30	554 (52.61)	0.301 (-1.008, 1.610)	0.249 (-1.127, 1.625)	0.110 (-1.297, 1.516)	1.397 (-0.125, 2.919)	0.3076	
Diabetes							0.2755
Diabetic	173 (16.43)	-0.076 (-2.928, 2.775)	2.174 (-0.554, 4.903)	-0.020 (-2.937, 2.898)	0.404 (-3.407, 4.215)	0.9786	
Not Diabetic	880 (83.57)	-0.402 (-1.266, 0.462)	0.288 (-0.618, 1.195)	-0.339 (-1.341, 0.663)	1.589 (0.555, 2.622)***	**0.0114**	
Hypertension							0.7503
Hypertensive	481 (45.68)	0.468 (-0.720, 1.655)	0.998 (-0.235, 2.231)	0.097 (-1.331, 1.525)	2.699 (1.180, 4.218)***	0.0701	
Not Hypertensive	572 (54.32)	-0.987 (-2.397, 0.422)	0.272 (-1.227, 1.770)	-0.560 (-2.092, 0.971)	0.708 (-0.914, 2.331)	0.1284	
**Healthy Eating Index 2015**							
**Variables**	N (%)	Q1[1]	Q2[1]	Q3[1]	Q4[1]	P_{Trend}[2]	$P_{Interaction}$[3]
Age							0.4323
≤ 50 years	594 (56.41)	0.487 (-0.756, 1.730)	1.038 (-0.304, 2.380)	1.711 (0.209, 3.213)*	2.442 (0.768, 4.116)***	**0.0183**	
> 50 years	459 (43.59)	-0.355 (-1.888, 1.177)	-0.139 (-1.608, 1.331)	-0.485 (-1.949, 0.979)	0.186 (-1.221, 1.592)	0.5627	
Sex							0.9239
Male	410 (38.94)	-0.440 (-1.784, 0.904)	-0.362 (-1.800, 1.075)	-0.415 (-1.950, 1.119)	1.560 (-0.213, 3.333)	0.0949	
Female	643 (61.06)	0.516 (-0.812, 1.843)	1.166 (-0.185, 2.518)	1.883 (0.484, 3.282)**	1.774 (0.470, 3.079)**	0.0935	
Race							0.7948
White	723 (68.86)	1.408 (0.407, 2.410)**	1.720 (0.618, 2.823)***	2.350 (1.134, 3.566)***	2.637 (1.441, 3.833)***	0.0502	
Black	327 (31.14)	-1.330 (-3.439, 0.779)	-1.033 (-3.052, 0.986)	-1.485 (-3.446, 0.475)	-0.249 (-2.359, 1.860)	0.5115	
Education							0.7215
≤ HS / GED[4]	727 (69.17)	-2.259 (-3.313, -1.204)***	-1.582 (-2.711, -0.452)**	-0.781 (-2.047, 0.484)	-0.723 (-2.010, 0.564)	**0.0224**	
> HS / GED[4]	324 (30.83)	2.963 (1.186, 4.740)***	2.923 (1.074, 4.771)***	3.163 (1.407, 4.919)***	4.710 (2.861, 6.559)***	0.0909	
Employment Status							0.3778
Employed	695 (66.25)	0.221 (-1.018, 1.461)	0.911 (-0.407, 2.229)	0.810 (-0.567, 2.186)	1.631 (0.269, 2.993)*	0.0613	
Not Employed	354 (33.75)	-0.486 (-2.254, 1.282)	-0.435 (-2.280, 1.411)	0.676 (-1.136, 2.488)	0.971 (-0.831, 2.772)	0.1837	

Smoking Status							0.9798
Active Smoker	177 (16.81)	0.645 (-1.312, 2.602)	0.390 (-1.399, 2.179)	2.994 (0.307, 5.682)*	4.106 (0.115, 8.097)*	**0.0392**	
Not Active Smoker	876 (83.19)	-0.130 (-1.247, 0.988)	0.470 (-0.668, 1.608)	0.430 (-0.634, 1.494)	1.160 (0.154, 2.166)*	0.0642	
Total Energy Intake							0.4305
≤ 2700 kcal/d	799 (75.88)	-0.222 (-0.993, 0.550)	0.615 (-0.157, 1.388)	1.046 (0.263, 1.830)**	0.699 (-0.063, 1.462)	**0.0396**	
> 2700 kcal/d	254 (24.12)	-0.207 (-1.613, 1.199)	0.006 (-1.419, 1.430)	0.443 (-1.023, 1.909)	1.556 (-0.003, 3.114)	0.0548	
Physical Activity[5]							0.4590
Low	622 (59.07)	-0.324 (-1.428, 0.780)	-1.081 (-2.196, 0.034)	0.076 (-1.105, 1.257)	-0.414 (-1.559, 0.732)	0.7801	
Moderate	113 (10.73)	-0.978 (-3.034, 1.079)	2.428 (0.245, 4.611)	-0.629 (-2.914, 1.65)	-0.077 (-2.752, 2.599)	0.5444	
High	318 (30.20)	-0.822 (-2.306, 0.662)	0.298 (-1.422, 2.018)	-0.038 (-1.567, 1.492)	0.748 (-0.966, 2.463)	0.1795	
BMI[6]							0.9829
< 30	499 (47.39)	-0.370 (-1.680, 0.941)	0.090 (-1.472, 1.651)	0.119 (-1.502, 1.740)	1.462 (-0.082, 3.006)	**0.0371**	
≥ 30	554 (52.61)	0.162 (-1.266, 1.590)	0.274 (-1.017, 1.564)	0.799 (-0.574, 2.173)	0.636 (-0.855, 2.126)	0.4865	
Diabetes							0.1422
Diabetic	173 (16.43)	1.594 (-1.475, 4.662)	-0.929 (-3.743, 1.885)	2.147 (-0.747, 5.040)	1.402 (-1.866, 4.669)	0.9873	
Not Diabetic	880 (83.57)	-0.486 (-1.367, 0.395)	0.268 (-0.639, 1.175)	0.163 (-0.843, 1.170)	1.292 (0.253, 2.331)*	**0.0098**	
Hypertension							0.6206
Hypertensive	481 (45.68)	0.071 (-1.173, 1.314)	1.248 (0.010, 2.487)*	0.727 (-0.637, 2.092)	2.287 (0.757, 3.818)***	**0.0259**	
Not Hypertensive	572 (54.32)	-0.523 (-1.962, 0.915)	-0.974 (-2.524, 0.575)	0.123 (-1.504, 1.750)	0.230 (-1.271, 1.730)	0.2626	
**Alternate Mediterranean Diet**							
**Variables**	N (%)	Q1[1]	Q2[1]	Q3[1]	Q4[1]	P_{Trend}[2]	$P_{Interaction}$[3]
Age							0.1228
≤ 50 years	594 (56.41)	0.408 (-1.133, 1.949)	0.905 (-0.696, 2.505)	1.163 (-0.075, 2.400)	1.800 (0.283, 3.318)*	0.1399	
> 50 years	459 (43.59)	-1.100 (-2.940, 0.740)	0.228 (-1.650, 2.105)	-0.219 (-1.434, 0.996)	0.344 (-1.080, 1.767)	0.2534	
Sex							0.3135
Male	410 (38.94)	-0.937 (-2.565, 0.691)	-0.194 (-1.857, 1.469)	-0.271 (-1.503, 0.961)	1.729 (-0.026, 3.484)	**0.0181**	
Female	643 (61.06)	0.443 (-1.187, 2.073)	1.704 (0.028, 3.380)*	1.368 (0.207, 2.529)*	1.513 (0.264, 2.762)*	0.4249	
Race							0.6041
White	723 (68.86)	1.001 (-0.161, 2.163)	2.328 (0.998, 3.657)***	1.944 (0.979, 2.908)***	2.638 (1.463, 3.813)	0.0606	
Black	327 (31.14)	0.108 (-3.037, 3.253)	-1.770 (-4.184, 0.64)	-1.193 (-2.810, 0.423)	-0.558 (-2.533, 1.416)	0.7387	
Education							0.4067
≤ HS / GED[4]	727 (69.17)	-2.286 (-3.643, -0.929)***	-1.569 (-2.854, -0.284)*	-1.505 (-2.499, -0.512)***	-0.716 (-1.968, 0.537)	0.0814	
> HS / GED[4]	324 (30.83)	2.245 (0.151, 4.340)*	4.132 (1.589, 6.657)***	3.048 (1.587, 4.510)***	4.486 (2.678, 6.294)***	0.0796	
Employment Status							0.2583
Employed	695 (66.25)	0.488 (-0.962, 1.938)	1.304 (-0.218, 2.827)	0.570 (-0.582, 1.722)	1.287 (-0.069, 2.642)	0.4398	
Not Employed	354 (33.75)	-1.889 (-4.117, 0.338)	-0.760 (-2.838, 1.317)	0.296 (-1.121, 1.714)	1.299 (-0.392, 2.989)	**0.0178**	
Smoking Status							0.8401
Active Smoker	177 (16.81)	-0.364 (-2.702, 1.973)	1.692 (-0.796, 4.180)	0.631 (-1.249, 2.511)	0.908 (-2.023, 3.839)	0.4529	
Not Active Smoker	876 (83.19)	-0.244 (-1.651, 1.163)	0.218 (-1.126, 1.561)	0.390 (-0.495, 1.275)	1.106 (0.107, 2.106)*	0.0668	
Total Energy Intake							0.4669
≤ 2700 kcal/d	799 (75.88)	-0.232 (-0.970, 0.506)	0.655 (-0.184, 1.494)	0.827 (0.140, 1.514)*	0.955 (0.058, 1.853)*	**0.0126**	
> 2700 kcal/d	254 (24.12)	-0.071 (-1.978, 1.837)	0.070 (-1.686, 1.827)	0.043 (-1.211, 1.297)	1.078 (-0.266, 2.422)	0.1759	
Physical Activity[5]							0.6765
Low	622 (59.07)	-0.163 (-1.549, 1.222)	-0.495 (-1.882, 0.892)	-0.693 (-1.656, 0.271)	-0.210 (-1.303, 0.883)	0.8744	
Moderate	113 (10.73)	-1.047 (-3.986, 1.892)	0.588 (-1.927, 3.102)	0.174 (-1.900, 2.247)	1.408 (-1.251, 4.066)	0.2231	
High	318 (30.20)	-0.533 (-2.324, 1.259)	-0.386 (-2.109, 1.337)	-0.106 (-1.447, 1.236)	0.463 (-1.127, 2.053)	0.3064	
BMI[6]							0.1828
< 30	499 (47.39)	-0.378 (-1.942, 1.186)	-0.149 (-1.780, 1.482)	-0.087 (-1.397, 1.224)	1.504 (0.012, 2.997)*	**0.0229**	
≥ 30	554 (52.61)	-0.119 (-1.805, 1.568)	0.879 (-0.888, 2.646)	0.625 (-0.508, 1.758)	0.401 (-1.032, 1.833)	0.8329	
Diabetes							0.4442
Diabetic	173 (16.43)	1.907 (-2.535, 6.348)	0.636 (-3.813, 5.085)	0.507 (-1.740, 2.753)	0.981 (-2.128, 4.090)	0.9095	
Not Diabetic	880 (83.57)	-0.657 (-1.707, 0.394)	0.321 (-0.775, 1.417)	0.203 (-0.600, 1.007)	1.033 (0.016, 2.049)*	**0.0271**	
Hypertension							0.6149
Hypertensive	481 (45.68)	-0.112 (-1.747, 1.522)	1.314 (-0.204, 2.831)	0.465 (-0.658, 1.588)	2.223 (0.863, 3.584)***	**0.0379**	
Not Hypertensive	572 (54.32)	-0.918 (-2.575, 0.740)	-0.649 (-2.518, 1.220)	-0.057 (-1.360, 1.246)	0.054 (-1.469, 1.576)	0.2836	

Association between Diet Quality and Cognitive Function in Midlife Adults: the Bogalusa Heart Study, Kristen Ogarrio

[1] Least squares mean estimate (95% CI). Adjusted for dietary quartile, age, sex, race, employment, education, smoking status, total energy intake, MET-minutes/week, BMI, diabetes, and hypertension
[2] *p*-value for linear trend calculated using the median from each quartile and treating it as a continuous variable
[3] *p*-value for interaction calculated by multiplying variable by continuous dietary score
[4] HS: High school; GED: General educational development
[5] Physical activity measured in MET-minutes/week according to IPAQ long form criteria
[6] BMI: Body mass index (kg/m^2)
$^{*}p < 0.05$ $^{**}p < 0.01$ $^{***}p < 0.005$

Association between Diet Quality and Cognitive Function in Midlife Adults: the Bogalusa Heart Study, Kristen Ogarrio

Supplementary Table S2. Stratified Adjusted Multinomial Odds Ratio (95% CI) of having Low Cognition Across Dietary Quartiles (N = 1053)

Alternate Healthy Eating Index 2010

Variables	N (%)	Q1[1]	Q2[2]	Q3[2]	Q4[2]	P_{Trend}[3]	Per SD Increase	$P_{Interaction}$[4]
Age								0.1129
≤ 50 years	594 (56.41)	1.000	0.602 (0.205, 1.768)	0.611 (0.155, 2.414)	0.676 (0.130, 3.526)	0.3606	0.808 (0.491, 1.330)	
> 50 years	459 (43.59)	1.000	0.167 (0.029, 0.964)*	0.687 (0.143, 3.289)	0.071 (0.012, 0.414)***	**0.0207**	0.495 (0.279, 0.876)†	
Sex								0.3449
Male	410 (38.94)	1.000	0.359 (0.080, 1.610)	1.458 (0.364, 5.841)	0.107 (0.016, 0.711)*	0.1761	0.689 (0.393, 1.210)	
Female	643 (61.06)	1.000	0.423 (0.136, 1.316)	0.376 (0.085, 1.655)	0.264 (0.065, 1.078)	**0.0437**	0.633 (0.391, 1.024)	
Race								0.3255
White	723 (68.86)	1.000	0.618 (0.217, 1.756)	0.790 (0.256, 2.438)	0.256 (0.057, 1.143)	0.0996	0.682 (0.433, 1.075)	
Black	327 (31.14)	1.000	0.960 (0.134, 6.865)	3.422 (0.358, 32.713)	0.264 (0.031, 2.219)	0.4955	0.739 (0.362, 1.511)	
Education								0.1506
≤ HS / GED[5]	727 (69.17)	1.000	0.395 (0.152, 1.028)	0.655 (0.224, 1.917)	0.185 (0.052, 0.658)**	**0.0231**	0.650 (0.436, 0.968)†	
> HS / GED[5]	324 (30.83)	1.000	0.770 (0.029, 20.275)	4.747 (0.249, 90.520)	0.890 (0.034, 23.354)	0.9129	1.137 (0.421, 3.073)	
Employment Status								0.2753
Employed	695 (66.25)	1.000	0.470 (0.137, 1.613)	0.920 (0.258, 3.285)	0.184 (0.043, 0.791)*	0.0587	0.653 (0.408, 1.046)	
Not Employed	354 (33.75)	1.000	0.356 (0.078, 1.624)	0.408 (0.080, 2.070)	0.114 (0.016, 0.830)*	0.0507	0.562 (0.290, 1.090)	
Smoking Status								0.0946
Active Smoker	177 (16.81)	1.000	4.226 (1.055, 16.921)*	1.858 (0.444, 7.773)	1.502 (0.226, 9.974)	0.4342	1.014 (0.960, 1.072)	
Not Active Smoker	876 (83.19)	1.000	0.412 (0.225, 0.755)***	0.735 (0.397, 1.359)	0.271 (0.137, 0.537)***	**0.0032**	0.970 (0.948, 0.992)††	
Total Energy Intake								0.7258
≤ 2700 kcal/d	799 (75.88)	1.000	0.573 (0.269, 1.224)	0.749 (0.360, 1.558)	0.441 (0.202, 0.962)*	0.0750	0.726 (0.545, 0.966)†	
> 2700 kcal/d	254 (24.12)	1.000	0.699 (0.217, 2.249)	1.051 (0.272, 4.070)	0.234 (0.049, 1.116)	0.1264	0.736 (0.457, 1.184)	
Physical Activity[6]								0.6794
Low	622 (59.07)	1.000	0.618 (0.325, 1.173)	1.701 (0.858, 3.373)	0.579 (0.290, 1.154)	0.5273	0.884 (0.695, 1.124)	
Moderate	113 (10.73)	1.000	2.228 (0.210, 23.639)	1.639 (0.29, 8.987)	1.538 (0.202, 11.719)	0.8595	1.061 (0.543, 2.072)	
High	318 (30.20)	1.000	0.589 (0.264, 1.313)	0.340 (0.132, 0.878)*	0.416 (0.150, 1.153)	**0.0357**	0.784 (0.556, 1.106)	
BMI[7]								0.5671
< 30	499 (47.39)	1.000	0.385 (0.095, 1.561)	0.764 (0.179, 3.267)	0.075 (0.012, 0.484)**	**0.0199**	0.469 (0.260, 0.846)†	
≥ 30	554 (52.61)	1.000	0.608 (0.182, 2.028)	1.113 (0.292, 4.237)	0.328 (0.079, 1.369)	0.2391	0.828 (0.513, 1.336)	
Diabetes								0.9511
Diabetic	173 (16.43)	1.000	0.257 (0.016, 4.205)	1.180 (0.112, 12.429)	0.865 (0.056, 13.349)	0.8917	1.030 (0.349, 3.041)	
Not Diabetic	880 (83.57)	1.000	0.530 (0.205, 1.368)	0.963 (0.339, 2.740)	0.168 (0.049, 0.578)***	**0.0203**	0.668 (0.455, 0.980)†	
Hypertension								0.3694
Hypertensive	481 (45.68)	1.000	0.437 (0.124, 1.546)	1.132 (0.260, 4.937)	0.058 (0.008, 0.414)***	**0.0196**	0.562 (0.321, 0.984)†	
Not Hypertensive	572 (54.32)	1.000	0.603 (0.170, 2.142)	0.881 (0.247, 3.141)	0.451 (0.105, 1.937)	0.3890	0.770 (0.478, 1.241)	

Healthy Eating Index 2015

Variables	N (%)	Q1[1]	Q2[2]	Q3[2]	Q4[2]	P_{Trend}[3]	Per SD Increase	$P_{Interaction}$[4]
Age								0.8410
≤ 50 years	594 (56.41)	1.000	0.395 (0.126, 1.237)	0.324 (0.083, 1.265)	0.180 (0.033, 0.972)*	**0.0356**	0.552 (0.324, 0.941)†	
> 50 years	459 (43.59)	1.000	0.849 (0.165, 4.368)	0.203 (0.039, 1.061)	0.372 (0.084, 1.653)	0.1352	0.647 (0.378, 1.108)	
Sex								0.2448
Male	410 (38.94)	1.000	1.520 (0.398, 5.807)	1.140 (0.271, 4.800)	0.397 (0.082, 1.930)	0.3393	0.679 (0.383, 1.205)	
Female	643 (61.06)	1.000	0.207 (0.056, 0.760)*	0.086 (0.019, 0.387)***	0.236 (0.060, 0.929)*	**0.0287**	0.575 (0.355, 0.931)†	
Race								0.1758
White	723 (68.86)	1.000	0.722 (0.259, 2.015)	0.335 (0.094, 1.186)	0.361 (0.105, 1.236)	0.0536	0.577 (0.370, 0.901)†	
Black	327 (31.14)	1.000	0.469 (0.062, 3.534)	0.169 (0.021, 1.382)	0.801 (0.064, 9.983)	0.5193	0.767 (0.361, 1.630)	
Education								0.2676
≤ HS / GED[5]	727 (69.17)	1.000	0.688 (0.372, 1.275)	0.344 (0.171, 0.693)***	0.466 (0.226, 0.963)*	**0.0090**	0.962 (0.936, 0.989)††	
> HS / GED[5]	324 (30.83)	1.000	0.486 (0.156, 1.509)	0.919 (0.284, 2.977)	0.583 (0.174, 1.956)	0.4872	0.966 (0.919, 1.015)	
Employment Status								**0.0143**
Employed	695 (66.25)	1.000	0.435 (0.129, 1.471)	0.338 (0.081, 1.411)	0.499 (0.132, 1.892)	0.2611	0.779 (0.484, 1.254)	
Not Employed	354 (33.75)	1.000	1.066 (0.154, 7.388)	0.188 (0.036, 0.968)*	0.107 (0.017, 0.678)*	**0.0096**	0.342 (0.170, 0.691)†††	

Smoking Status								0.9683
Active Smoker	177 (16.81)	1.000	1.110 (0.330, 3.672)	0.639 (0.158, 2.585)	0.245 (0.027, 2.272)	0.2703	0.964 (0.903, 1.028)	
Not Active Smoker	876 (83.19)	1.000	0.622 (0.338, 1.146)	0.441 (0.229, 0.848)*	0.505 (0.265, 0.963)*	**0.0283**	0.967 (0.942, 0.991)††	
Total Energy Intake								0.8719
≤ 2700 kcal/d	799 (75.88)	1.000	0.577 (0.276, 1.204)	0.340 (0.154, 0.751)**	0.486 (0.224, 1.051)*	**0.0389**	0.823 (0.625, 1.083)	
> 2700 kcal/d	254 (24.12)	1.000	0.786 (0.233, 2.653)	0.465 (0.125, 1.734)	0.395 (0.088, 1.772)	0.1775	0.599 (0.355, 1.010)	
Physical Activity[6]								0.9587
Low	622 (59.07)	1.000	1.006 (0.529, 1.913)	0.946 (0.478, 1.873)	0.989 (0.508, 1.923)	0.9874	0.939 (0.741, 1.190)	
Moderate	113 (10.73)	1.000	0.080 (0.011, 0.609)*	0.279 (0.042, 1.838)	1.504 (0.182, 12.436)	0.8103	0.894 (0.446, 1.790)	
High	318 (30.20)	1.000	0.492 (0.198, 1.220)	0.328 (0.132, 0.816)*	0.551 (0.192, 1.579)	0.1229	0.731 (0.502, 1.064)	
BMI[7]								0.5155
< 30	499 (47.39)	1.000	0.557 (0.139, 2.241)	0.387 (0.078, 1.926)	0.175 (0.035, 0.886)*	**0.0255**	0.431 (0.235, 0.793)††	
≥ 30	554 (52.61)	1.000	0.455 (0.137, 1.506)	0.244 (0.067, 0.894)*	0.748 (0.177, 3.163)	0.3146	0.785 (0.485, 1.273)	
Diabetes								0.7921
Diabetic	173 (16.43)	1.000	1.207 (0.104, 13.994)	0.334 (0.023, 4.916)	0.761 (0.064, 9.025)	0.6694	0.878 (0.346, 2.230)	
Not Diabetic	880 (83.57)	1.000	0.505 (0.192, 1.328)	0.321 (0.108, 0.949)*	0.352 (0.114, 1.085)	**0.0346**	0.600 (0.404, 0.891)†	
Hypertension								0.3036
Hypertensive	481 (45.68)	1.000	0.192 (0.051, 0.728)*	0.188 (0.043, 0.826)*	0.119 (0.020, 0.705)*	**0.0091**	0.487 (0.277, 0.853)†	
Not Hypertensive	572 (54.32)	1.000	1.438 (0.407, 5.077)	0.466 (0.110, 1.968)	0.779 (0.198, 3.067)	0.4897	0.734 (0.444, 1.213)	
**Alternate Mediterranean Diet**								
Variables	N (%)	Q1[1]	Q2[2]	Q3[2]	Q4[2]	P_{Trend}[3]	Per SD Increase	$P_{Interaction}$[4]
Age								0.1728
≤ 50 years	594 (56.41)	1.000	0.468 (0.107, 2.051)	0.538 (0.159, 1.818)	0.301 (0.054, 1.669)	0.2068	0.672 (0.398, 1.134)	
> 50 years	459 (43.59)	1.000	0.451 (0.049, 4.175)	0.198 (0.030, 1.295)	0.102 (0.014, 0.763)*	**0.0160**	0.448 (0.244, 0.823)††	
Sex								0.6150
Male	410 (38.94)	1.000	0.960 (0.164, 5.629)	1.009 (0.243, 4.197)	0.163 (0.024, 1.120)	0.0731	0.587 (0.325, 1.061)	
Female	643 (61.06)	1.000	0.184 (0.037, 0.923)	0.168 (0.040, 0.706)*	0.165 (0.034, 0.791)*	0.0816	0.576 (0.343, 0.965)†	
Race								0.1133
White	723 (68.86)	1.000	0.498 (0.134, 1.841)	0.352 (0.118, 1.051)	0.191 (0.048, 0.753)*	**0.0156**	0.530 (0.335, 0.836)††	
Black	327 (31.14)	1.000	0.389 (0.016, 9.534)	1.216 (0.080, 21.190)	0.550 (0.024, 12.784)	0.9519	1.111 (0.443, 2.784)	
Education								0.8779
≤ HS / GED[5]	727 (69.17)	1.000	0.491 (0.143, 1.688)	0.462 (0.157, 1.363)	0.235 (0.064, 0.865)*	**0.0343**	0.616 (0.404, 0.941)†	
> HS / GED[5]	324 (30.83)	1.000	0.811 (0.025, 26.613)	0.422 (0.024, 7.460)	0.165 (0.005, 5.287)	0.2394	0.530 (0.179, 1.574)	
Employment Status								0.1100
Employed	695 (66.25)	1.000	0.832 (0.171, 4.054)	0.721 (0.191, 2.721)	0.475 (0.103, 2.198)	0.3159	0.691 (0.423, 1.127)	
Not Employed	354 (33.75)	1.000	0.178 (0.020, 1.555)	0.156 (0.023, 1.068)	0.037 (0.004, 0.370)***	**0.0056**	0.374 (0.182, 0.771)††	
Smoking Status								0.4514
Active Smoker	177 (16.81)	1.000	0.141 (0.029, 0.677)*	0.788 (0.232, 2.683)	0.542 (0.80, 3.665)	0.7405	0.886 (0.646, 1.217)	
Not Active Smoker	876 (83.19)	1.000	0.890 (0.399, 1.985)	0.570 (0.289, 1.125)	0.361 (0.168, 0.775)**	**0.0029**	0.814 (0.711, 0.933)†††	
Total Energy Intake								0.8035
≤ 2700 kcal/d	799 (75.88)	1.000	0.581 (0.626, 1.271)	0.384 (0.195, 0.756)**	0.387 (0.168, 0.890)*	**0.0122**	0.685 (0.519, 0.905)††	
> 2700 kcal/d	254 (24.12)	1.000	0.650 (0.126, 3.360)	0.771 (0.185, 3.210)	0.242 (0.045, 1.306)	0.0658	0.604 (0.350, 1.041)	
Physical Activity[6]								0.7726
Low	622 (59.07)	1.000	0.931 (0.413, 2.099)	1.168 (0.580, 2.355)	0.808 (0.379, 1.723)	0.5807	0.897 (0.698, 1.153)	
Moderate	113 (10.73)	1.000	0.379 (0.035, 4.084)	0.145 (0.018, 1.146)	0.175 (0.016, 1.927)	0.2184	0.590 (0.268, 1.296)	
High	318 (30.20)	1.000	0.858 (0.306, 2.408)	0.469 (0.189, 1.164)	0.386 (0.137, 1.090)	**0.0479**	0.759 (0.536, 1.073)	
BMI[7]								0.3820
< 30	499 (47.39)	1.000	1.120 (0.178, 7.057)	0.412 (0.097, 1.748)	0.081 (0.012, 0.534)**	**0.0044**	0.390 (0.217, 0.702)†††	
≥ 30	554 (52.61)	1.000	0.309 (0.066, 1.456)	0.518 (0.135, 1.988)	0.388 (0.080, 1.878)	0.4464	0.876 (0.513, 1.496)	
Diabetes								0.9396
Diabetic	173 (16.43)	1.000	0.108 (0.002, 6.561)	0.286 (0.012, 7.058)	0.277 (0.009, 8.363)	0.7504	0.736 (0.236, 2.288)	
Not Diabetic	880 (83.57)	1.000	0.474 (0.141, 1.595)	0.429 (0.152, 1.207)	0.176 (0.049, 0.629)**	**0.0088**	0.565 (0.376, 0.850)††	
Hypertension								0.3169
Hypertensive	481 (45.68)	1.000	0.199 (0.034, 1.177)	0.427 (0.086, 2.125)	0.027 (0.003, 0.227)***	**0.0019**	0.426 (0.226, 0.804)††	
Not Hypertensive	572 (54.32)	1.000	1.070 (0.207, 5.518)	0.389 (0.111, 1.358)	0.527 (0.121, 2.294)	0.3128	0.668 (0.411, 1.088)	

[1] Reference category

Association between Diet Quality and Cognitive Function in Midlife Adults: the Bogalusa Heart Study, Kristen Ogarrio

[2] Odds ratio (95% CI). Adjusted for dietary quartile, age, sex, race, employment, education, smoking status, total energy intake, MET-minutes/week, BMI, diabetes, and hypertension
[3] *p*-value for linear trend calculated using the median from each quartile and treating it as a continuous variable
[4] *p*-value for interaction calculated by multiplying variable by continuous dietary score
[5] HS: High school; GED: General educational development
[6] Physical activity measured in MET-minutes / week according to IPAQ long form criteria
[7] BMI: Body mass index (kg/m^2)

$^{*}p < 0.05$ $^{**}p < 0.01$ $^{***}p < 0.005$
$^{\dagger}p < 0.05$ $^{\dagger\dagger}p < 0.01$ $^{\dagger\dagger\dagger}p < 0.005$
